# COVID-19 Induced Economic Slowdown and Mental Health Issues

**DOI:** 10.3389/fpsyg.2022.777350

**Published:** 2022-03-04

**Authors:** Yimiao Gong, Xiaoxing Liu, Yongbo Zheng, Huan Mei, Jianyu Que, Kai Yuan, Wei Yan, Le Shi, Shiqiu Meng, Yanping Bao, Lin Lu

**Affiliations:** ^1^Peking University Sixth Hospital, Peking University Institute of Mental Health, NHC Key Laboratory of Mental Health (Peking University), National Clinical Research Center for Mental Disorders (Peking University Sixth Hospital), Beijing, China; ^2^Peking-Tsinghua Centre for Life Sciences and PKU-IDG/McGovern Institute for Brain Research, Peking University, Beijing, China; ^3^National Institute on Drug Dependence and Beijing Key Laboratory of Drug Dependence, Peking University, Beijing, China; ^4^School of Public Health, Peking University, Beijing, China

**Keywords:** COVID-19, economic slowdown, mental health issues, GDP, international trade, unemployment, healthcare system, education system

## Abstract

The COVID-19 pandemic has pressed a pause button on global economic development, and induced significant mental health problems. In order to demonstrate the progressed relationship between the pandemic, economic slowdown, and mental health burden, we overviewed the global-level gross domestic product changes and mental problems variation since the outbreak of COVID-19, and reviewed comprehensively the specific sectors influenced by the pandemic, including international trade, worldwide travel, education system, healthcare system, and individual employment. We hope to provide timely evidence to help with the promotion of policymakers’ effective strategies in mitigating economic losses induced by the pandemic; we suggest different governments or policy makers in different countries to share information and experience in dealing with COVID-19-induced economic slowdown and promote COVID-19 vaccine popularization plan to protect every individual worldwide against the coronavirus essentially; and we appeal international information share and collaboration to minimize stigmatization related to adverse mental consequences of COVID-19 and to increase mental health wellbeings of people all over the world.

## Introduction

Since the first outbreak of COVID-19 caused by Severe Acute Respiratory Syndrome Coronavirus 2 at the end of 2019, the coronavirus has been keeping hovering all over the world and depressing the world populations’ lives like a huge mist. With tens of thousands of individuals infected worldwide, medical emergency systems activated, and individuals’ normal lives damaged, COVID-19 has pressed a pause button on economic development and induced significant mental health problems (Cross et al., 2020; Guan et al., 2020; Keogh-Brown et al., 2020; Sarkodie and Owusu, 2020). Right following outbreak, some researchers started paying attention to the impact of the pandemic on economic loss, and many others focused on investigating the influence on mental health burden. However, it is important to learn about the interrelation of the three on the process of establishing and implementing cost-effect strategies to fight against the pandemic and the plight related to COVID-19. Thus, in this review article, we aimed to present the connection between the three – we overviewed the changes of global-level gross domestic product (GDP) and variation in mental problems since the outbreak of COVID-19, reviewed comprehensively the specific sectors influenced by the pandemic, and looked at the role that cultural and national differences might play in this relationship.

## Overview of the Effect of COVID-19 on Economic and Mental Health

**FIGURE 1:**
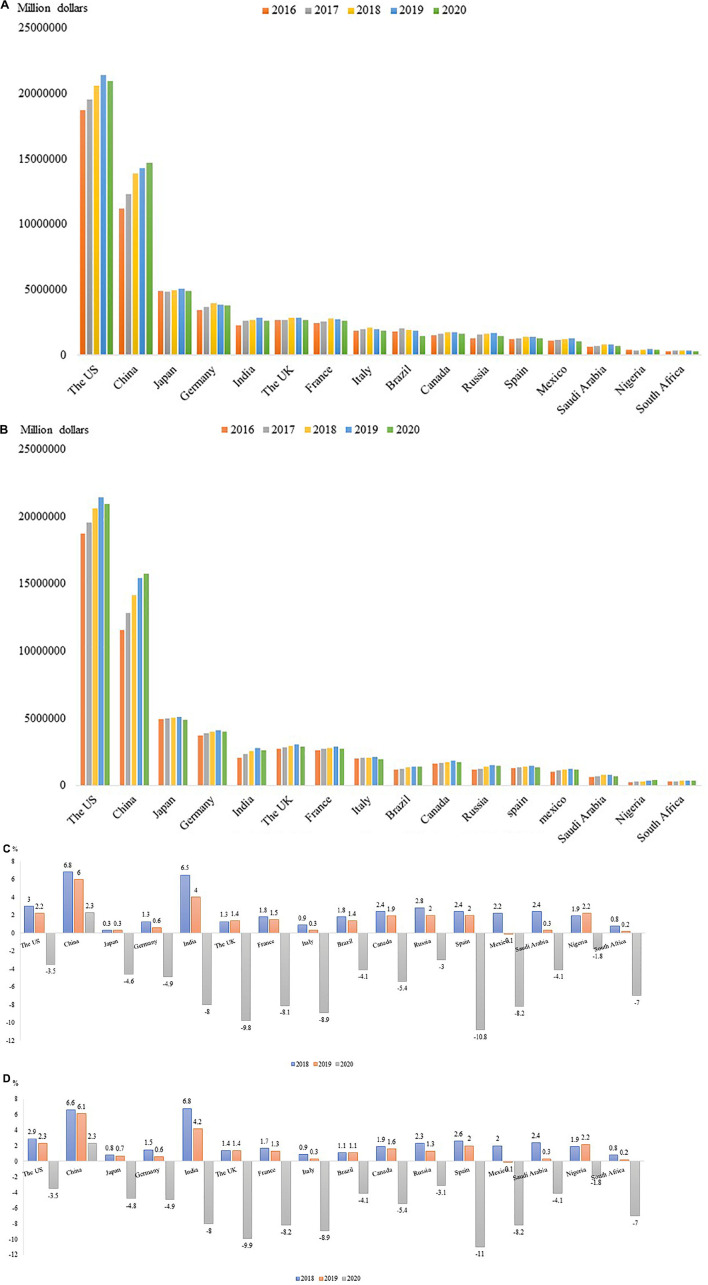
**(A)** GDP Changes from 2016 to 2020 (World Bank). Based on data from the World Bank database, the change in GDP of the world’s major economies from 2016 to 2020 is calculated in current US dollars (August 21, 2021). The World Bank database lacks the GDP data of Japan in 2020. The data here comes from the Statistics of the Japanese Cabinet Office and is converted to current US dollars (Japanese Cabinet Office: https://www.esri.cao.go.jp/jp/sna/menu.html). **(B)** GDP Changes from 2016 to 2020 (IMF). Based on data from International Monetary Fund (IMF) database, the change in GDP of the world’s major economies from 2016 to 2020 is calculated in current US dollars (September 8, 2021). The IMF database lacks 2020 GDP data for China, India, Russia and Nigeria, which are derived from the World Bank database and converted to current US dollars. **(C)** Changes in annual GDP growth (%) rates in major economies (World Bank). Based on data from the World Bank database. The World Bank database lacks the GDP data of Japan in 2020. The data here comes from the Statistics of the Japanese Cabinet Office (Japanese Cabinet Office: https://www.esri.cao.go.jp/jp/sna/menu.html). **(D)** Changes in annual GDP growth (%) rates in major economies (IMF).

Economic activities are easily affected by various social factors, such as monetary policies (Balke et al., 2017; Zhang, 2019; An et al., 2021), credit shocks (Balke et al., 2021), and major public events like COVID-19 pandemic (Shang et al., 2021; Zhao Y. H. et al., 2021). The global economy encountered a serious recession in the second quarter of 2020. To demonstrate the economic changes more clearly, we summarized recent 5-year GDP changes in 16 countries worldwide and recent 3-year changes in GDP growth rates in major economies according to The World Bank (2021), International Monetary Fund (IMF) (2021), and Japanese Cabinet Office (National Accounts of Japan, 2021) (Figures 1A–D).

For most demonstrated countries, GDP increased between 2016 and 2019, whereas the increasing trend stopped abruptly in 2019 (Figures 1A,B). Negative GDP growth rates were encountered by most countries in 2020, except for China, based on World Bank and IMF estimations (Figures 1C,D). Although a causal relationship or direct association between economic shrink and COVID-19 cannot be concluded through the data, the pandemic played at least some roles in the economic crisis since 2019 and 2020 were the years when COVID-19 outbroke. In terms of a World Bank estimation, COVID-19 pandemic pushed between 88 million and 115 million people into extreme poverty in 2020. A research team from Italy used electricity market data to monitor real-time economic impact of the containment policies. According to their estimation, the 3 weeks of the most repressive outbreak period in Italy reduced the corresponding national GDP by about 30% (Fezzi and Fanghella, 2020). Another research suggested that the estimated GDP loss caused by COVID-19 related unemployment had reached 7.6 trillion, and the overall estimated GDP decline resulted from the pandemic was 3.4% (Arredondo et al., 2021). China, as the only country with no negative GDP growth, also encountered a huge challenge in the economic development due to COVID-19. For example, Zhao J. et al. (2021) estimated the economic burden of movement restriction policies (MRPs) and found that the first wave of COVID-19 in China resulted in a cost of 278 billion USD. Another Chinese team investigated different aspects of economic costs more carefully and concluded that although healthcare costs including inpatient care costs and other medicines costs account for a major part of economic burden in China, productivity losses which were mostly attributable to the effect of MRPs on people who did not have COVID-19 accounted for 99.8% of societal costs. Moreover, the control measures used to prevent COVID-19 spread have resulted in huge productivity losses which amounted to 2.7% (US$ 382.29 billion/US$ 14.14 trillion) of China’s annual GDP (Jin et al., 2021).

Mental health changes related to COVID-19 outbreak have been investigated widely. Depression, anxiety, insomnia, acute stress or post-traumatic stress disorder (PTSD), suicide behaviors are common mental health problems which individuals suffer during COVID-19 (Bao et al., 2020; Meng et al., 2020; Shi et al., 2020; Wang et al., 2020; Xu et al., 2021). Table 1 gives a summary of findings that explored variation of prevalence of mental health problems relative to the COVID-19 outbreak period. According to most studies, prevalence of mental problems was the highest during the outbreak period compared with both before outbreak and post outbreak (Barrea et al., 2020; Gallagher and Wetherell, 2020; Li X. Y. et al., 2020; McGinty et al., 2020; Salfi et al., 2020; van der Velden et al., 2020; Capuano et al., 2021; Creese et al., 2021; Jalal et al., 2021; Johnson et al., 2021; Martinez-de-Quel et al., 2021; Megalakaki et al., 2021; Mei et al., 2021; Pieh et al., 2021; Ramiz et al., 2021; Yang et al., 2021). One study from Argentine reported an even higher prevalence of sleep problems and moderate to severe depressive symptoms post outbreak (32.1 and 47.8%, respectively) compared with that during outbreak (23.1 and 24.3%, respectively). Shi et al. (2021a) from China reported a higher persistent prevalence of depression and insomnia (33.6 and 35.3% vs. 30 and 29.8%, respectively) from first wave of pandemic to the aftermath of the outbreak (Shi et al., 2021a). These reports suggested the huge adverse effect of COVID-19 outbreak on mental health conditions (Badellino et al., 2021).

**TABLE 1:** Summary of findings investigating prevalence of mental health problems relative to the COVID-19 outbreak period.

References	Site	Participants	Outcomes	Prevalence-Before	Prevalence-During	Prevalence-Post
Badellino et al. (2021)	Argentine	general population	bad sleep quality	/	23.1% (459/1985)	32.1% (912/2839)
Barrea et al. (2020)	Italy	general population	poor sleep quality	50.4% (61/121)	/	81% (98/121)
			moderate to severe depression	/	24.3% (482/1985)	47.8% (1357/2839)
Capuano et al. (2021)	Italy	patients with physical disorder	depression	11.90% (8/67)	11.90% (8/67)	/
			anxiety	11.90% (8/67)	16.40% (11/67)	/
Creese et al. (2021)	United Kingdom	older adults (age M 50)	mild depression	13.2% (392/3281)	19% (634/3281)	/
			moderate-to-severe depression	4.1% (124/3281)	5.6% (185/3281)	/
			mild anxiety	9.3% (276/3281)	12.6% (415/3281)	/
			moderate-to-severe anxiety	2.2% (66/3281)	2.7% (89/3281)	/
Gallagher and Wetherell (2020)	United Kingdom	caregivers	depression	16.7% (225/1349)	21.6% (291/1349)	/
		non-caregivers	depression	12.1% (748/6178)	17.9% (1106/6178)	/
Jalal et al. (2021)	Saudi Arabia	students studying bachelor’s degree programs	bad sleep quality	70.1% (440/628)	79.9% (502/628)	/
Johnson et al. (2021)	Norway	general population	depression	/	23% (659/2868)	16.8% (250/1489)
			anxiety	/	23.3% (667/2868)	13.8% (205/1489)
Li X. Y. et al. (2020)	Wuhan, China	anesthesiologists and operating room medical staffs	depression	/	41.6% (82/197)	13.2% (26/197)
			anxiety	/	43.1% (85/197)	15.7% (31/197)
Martinez-de-Quel et al. (2021)	Spain	general population	poor sleep quality	63.40% (102/161)	75.2% (121/161)	
McGinty et al. (2020)	United States	general population	stress	3.9% (991/25417)	13.6% (199/1468)	/
Mei et al. (2021)	China	pregnant	mild depression	20.5% (482/2352)	36.2% (192/531)	/
			moderate depression	0.6% (13/2352)	10.4% (55/531)	/
			mild anxiety	24.7% (580/2352)	26.9% (143/531)	/
			moderate anxiety	3.1% (73/2352)	4.5% (24/531)	/
Megalakaki et al. (2021)	France	general population	COVID-19 peritraumatic distress	/	35.5% (398/1123)	17.2% (40/232)
Pieh et al. (2021)	Austria	general population	moderate	/	14.6% (64/437)	15.6% (68/437)
			moderate to severe	/	18.3% (80/437)	19.7% (86/437)
			depression moderate anxiety	/	16.5% (72/437)	15.6% (68/437)
Ramiz et al. (2021)	France	general population	depression	27% (278/5356)	27.6% (341/1237)	/
			anxiety	17.3% (180/5476)	20.1% (248/1237)	/
Salfi et al. (2020)	Italy	woman	moderate to	/	13.12% (254/2701)	11.63%
			severe insomnia			(314/2701)
		man		/	9.37% (253/2701)	12.02%
						(324/2701)
		woman	depression	/	7.20% (194/2701)	6.50% (175/2701)
		man		/	4.47% (120/2701)	5.71% (154/2701)
Shi et al. (2021a)	China	general population	depression	/	30% (3151/10492)	33.6%
						(3528/10492)
			anxiety	/	35.2% (3693/10492)	32.5%
						(3415/10492)
			insomnia	/	29.8% (3127/10492)	35.3%
						(3701/10492)
			any mental health symptoms	/	46.4% (4865/10492)	45.1% (4733/10492)
van der Velden et al. (2020)	Netherlands	general population	anxiety and depression	7.2% (239/3983)	7.8% (257/2980)	/
Yang et al. (2021)	Hangzhou	general population	depression	13.7% (431/4144)	20.2% (636/3153)	/

**FIGURE 2:**
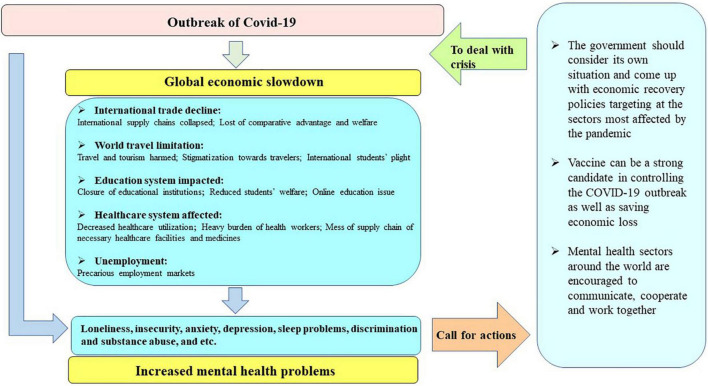
Summary flow chart.

The economic crisis triggered by COVID-19 pandemic can be considered as one possibility accounting for COVID-19-related mental health problems (McInerney et al., 2013). It is generally recognized that economic crisis has a vicious impact on mental health (Sun et al., 2020). One-month prevalence of major depression was found to be 8.2% in the Greek population in 2011, the year that the country was in the throes of economic collapse, which was 2.6 times compared to the prevalence rate in 2008 (Economou et al., 2013). Since 2007, nearly all European countries have encountered the economic shock and the situation was the worst in Spain. Compared with the pre-crisis period of 2006, the proportion of patients with mood, anxiety, somatoform, and alcohol-related disorders had a substantial and significant increase during the economic crisis period. Families experiencing unemployment and mortgage payment difficulties suffered the most (Gili et al., 2013). The October 2008 stock market crash in the United States had put a large number of older adults into depressive status and the adverse effect of sudden economic shock was the largest among individuals with high levels of stock holding prior to the crash. In the case of COVID-19, a sharp decline or negative growth of GDP is a macroeconomic reflection of the pandemic’s impact on per capita income (Capello and Caragliu, 2021). Individuals encountered job loss and/or income contraction due to the pandemic, which would contribute to their worse life quality and subjective wellbeing (Rasul et al., 2021). Therefore, in this review, we propose a progressive relationship between COVID-19, economic slowdown, and mental health problems (Figure 2). That is, we believe that the economic slowdown induced by COVID-19 pandemic is interrelated with many negative mental health consequences during this period. In the next part of this review, we will look into more details about the interrelationship between the three.

## Specific Sectors Impacted by COVID-19 and Related Mental Problems

In the following session, we list specific economic impacts of COVID-19 pandemic, including international trade, travel, education system, healthcare system, and individual employment, as well as mental health problems related to these economic impacts.

### International Trade Decline and Associated Mental Health Problems

COVID-19 created an international trade decline through reducing both demand and supply. For example, China is one of the biggest countries worldwide exporting manufactured goods, whereas, export prohibitions and restrictions introduced by over 80 countries and customs territory due to the pandemic blocked the just-in-time manufacturing between China and the other areas in the world and drove a huge chock on manufacturing market (Barlow et al., 2021). Estimated contraction of total global merchandise trade was by 9.2% in 2020, with a hard hit on supplies of pharmaceuticals and medical equipment, leading to detrimental consequences for physical and mental health (Barlow et al., 2021). Many global trades nowadays focus not on price the most but on relation the most. In the relation-driven agent-based global trade model, the role of relational factors including trust, familiarity, reputation, trade history and conflicts in countries’ trade behavior is very important. Healthy global trade relationship with close international interaction and communication has been suggested to improve the food and nutrition security of countries in Africa, Asia and Latin America and successively promote a healthier and more balanced diet (Ge et al., 2021). COVID-19 has a negative effect on the balance of the relation-driven global trade and the limited communication among countries will lead to a huge alternation of individual physical and mental wellbeing eventually.

Besides, as for individuals living in border areas and those who rely on international supply chains to earn lives, the collapse of many international supply chains is like a bolt from the blue. The UN World Food Programme estimated that by the end of 2020, the number of people suffering from severe hunger will almost double due to the pandemic. Theories of international trade propose that foreign trade can lead to a society’s ultimate gain in comparative advantage and welfare, which is significant for the social members’ mental health (Lin et al., 2020). The team of Lin et al. (2020) applied a fixed effects regression model to explore the prevalence of depressive disorders as a function of international trade and found that a 100% increase in the value of international trade indicated a 0.09%-point decrease in the prevalence of depressive disorders, and vice versa. Similarly, import competition which disproportionately brought wealth shock on industry labors could have a large negative impact on individual mental health, which can even have negative spillovers to other family members’ mental wellbeing (Colantone et al., 2019). Hence, the affected international trade by COVID-19 pandemic is not only an unfortune for global economic growth, but also for individual mental wellbeing.

### Worldwide Travel Limitation and Potential Mental Health Problems

The World Travel and Tourism Council warned that the outbreak of COVID-19 might put 50 million jobs in the global travel and tourism sector into risk, leaving the tourism sector as one of the hardest-hit due to the pandemic (Ataguba, 2020; Nicola et al., 2020; Siddiquei and Khan, 2020; Rasul et al., 2021). In South Asia, the travel and tourism sector created about 50 million jobs in 2018, which contributed to a significant amount of national GDP. However, the outbreak of COVID-19 and the mitigation measure drove demands in tourism and travel into collapse all of a sudden. Chinese visitors received by Vietnam dropped from about 1.45 million in the first quarter of 2019 by 644,000 in January of 2020, which resulted in a huge economic loss (Nicola et al., 2020). On March 12, 2020, the Nepal government canceled all Everest expeditions slated for the spring season, thus, expenses including US$4 million annual collections in royalties from Everest climbing permits and US$40,000–90,000 other expenses were in loss (Rasul et al., 2021). In the trekking sector alone in South Asia, approximately 20,000 tour guides would lose their jobs due to the tourism demand contraction, up to 60,000 employees in the hospitality sector in major capitals were affected, and 40–50 million job cuts were imminent from big hotels, travel agencies and tour operators in India (Rasul et al., 2021). Implement of travel ban compelled the cancelation of a number of airlines, such as Lufthansa and Cathay Pacific, Chinese airlines, Singapore Airlines, etc. Employees in these companies were asked to stay at home and there might be a possibility of layoffs due to the reduced number of flights (Siddiquei and Khan, 2020).

Travel should have been a wholesome activity for most people. On the trip, travelers from different countries and regions can exchange their unique cultures and happiness with each other through active communication and interaction. However, as governments across countries advised their citizens to avoid unnecessary travel, subjective stigmatization toward travelers was developed, especially toward those belonging to Coronavirus-prone countries. This feeling could be a culprit for further mental health problems among the population. For individuals who have traveled in the epidemic area before the outbreak, according to research, they were more likely to suffer from internalized stigmatization associated with the travel experiences. They may develop internal fear of discrimination and exclusion by other members of the community and further develop negative emotions and cognitions such as self-deprecation, feeling dirty, and self-blame, and in more severe cases, symptoms of anxiety and depression will make the traveler to suffer a lot (Li J. et al., 2020).

A special group of “travelers” is the team of international students who travel to another country with different cultures and local customs and practices, and who will return to their own countries after the learning experience. With the impact of COVID-19, many international students had to return to their home country or region and were unable to go back to school for classes. These students faced a wide range of transitional events and ongoing stressors as they had to adapt to new academic environments and demands in order to keep up with their studies. There are also international students unable to return to their families and friends in the home country due to travel restrictions and extremely high travel expenses. They are away from their central social support systems and worried about their family members’ conditions, all of which can be great challenges during the special period and hence affect their mental health (Lai et al., 2020). An investigation showed that 84.7% of the studied international students reported moderate-to-high perceived stress since outbreak of the pandemic, 12.1% had moderate-to-severe symptoms of anxiety and depression, and 17.1% had moderate-to-severe symptoms of insomnia (Lai et al., 2020). Feeling lonely is the main concern for international students who undergo a self-isolation process. Economic shock brought by the pandemic put international students’ lives in the host country in more difficulty since the price of commodities could be higher than usual. Students who are about to graduate could feel more stressed because of the unforeseen future including unknown graduate dates. Research showed that international students facing job-hunting, severe economic pressure, and poor self-rated health status had higher levels of stress, anxiety, depression, and PTSD symptoms (Song et al., 2021; Wilczewski et al., 2021). Many international Chinese students also experience discrimination and isolation in some countries due to being deemed as potential coronavirus carriers, which can lead to mental problems such as denial, stress, anxiety, and fear (Zhai and Du, 2020). Fortunately, social support was shown to be a protective factor for mental health wellbeing. More social interaction and communication will help keep individuals’ positive emotions (Li J. et al., 2020).

### Education System by COVID-19 and Associated Mental Health Problems

All levels of the education system, from preschool to tertiary education, has been affected by the outbreak of pandemic. Over 100 countries have imposed a nationwide closure of educational facilities, although different countries implemented various policies with some countries announcing complete closure like in Germany and Italy and other countries announcing targeted closure like in the United Kingdom. In the case of Australia, the higher education sector expected the revenue loss to be AUS$3–4.6 billion in the academic year 2019–2020. Besides, the number of international student enrollments in the United States was expected to reduce by at least a quarter, with a huge loss of income from the higher education sector (Thepphakorn et al., 2021). According to the estimation by United Nations Educational Scientific and Cultural Organization (UNESCO), up to 900 million learners were affected by the closure of educational institutions (Nicola et al., 2020). Many parents have to stay at home from work with their children out of school. The United States federal government put school closures as an important strategy for Pandemic Influenza Mitigation. Although effective in reducing virus transmission, previous literature showed that closing schools in the United States for 4 weeks could cost between $10 and $47 billion which accounted for 0.1–0.3% of GDP as well as a reduction of 6 to 19% in key health care personnel (Lempel et al., 2009). Similarly, in the United Kingdom, researchers have found that closing all schools for 4 weeks would cost between 0.1 and 0.4% of GDP and about 16% of the workforce would take absenteeism since they were the main caregiver of dependent children (Sadique et al., 2008). Schools were no longer able to provide free school meals for children which had a significant impact on childcare costs for families with young children, especially for low-income families. This added new household expenditure that could otherwise be economized (Alvi and Gupta, 2020). In addition, online learning has become a new fashion of education ushered by the pandemic. Just like a space of classroom as the necessity for face-to-face education, digital technologies and electronic devices are the new necessities for online education. This new normal of education strategy is always fueled by commercialism and the reigning market ideology, compared to the traditional education system. In fact, not all students were able to afford this internet-based economic activity (Pacheco, 2020; Muthuprasad et al., 2021; Rasul et al., 2021). What’s more, school closures as a short-term emergency measure to contain pandemic may lead to students’ increased dropout possibility from school in the long-term. Based on UNESCO estimation, about 0.32 billion students in India had been affected by school closures since the outbreak of COVID-19, many of whom will be pushed into the labor market without adequate education background (Alvi and Gupta, 2020).

A remote online learning strategy may cause a series of emotional issues for the learners. Unpreparedness to learn an online course, working with “unknown others,” unfamiliarity with information technology are all potential risk factors contributing to individuals’ anxiety. In the case of COVID-19, some specific anxious factors are the sudden absence of a scheduled university environment, an unprecedented shift from school to home environment, disrupted learning and classroom routines, increased academic workload, uncertainty about academic progress (Zhao T. et al., 2021). As students encounter a limited opportunity of communication with peers and teachers, they will suffer from the lack of socialization and the stressed feeling of having fallen academically behind other peers. Without proper intervention, these negative feelings can eventually give rise to comorbidities such as depressive symptoms (Li H. et al., 2020).

Hasan and Bao (2020) evaluated the psychological effects of COVID-19 “e-learning crack-up” with a sample of 400 higher education students in Bangladesh and concluded that fear of academic year loss was a great predictor for students’ psychological distress during the pandemic. Extreme feelings of seclusion, helplessness, panic attacks, and comorbid sleep problems have been often reported by student respondents during school closures due to the pandemic (Li H. et al., 2020; Shih et al., 2021; Tzafilkou et al., 2021). What’s worse is that study suggested lower educational attainment due to school closure was associated with lifelong mental health problems including but not limited to depression and schizophrenia (Yoshikawa et al., 2020). The mental health of university students was also being affected by abnormality of daily routine, such as lack of daily leisure and social interaction and loss of academic routines, which included classes and clinical sessions. In addition, students who were presented with history of mental illness and chronic medical illness should also be given additional attention as they are prone to develop mental health complications, such as depression and anxiety. A protective factor of utmost important was greater degree of social support received from friends and family members. In addition to the mental health impact, the sudden alteration in the conduct of education in university also lowered the psychological and social aspects of quality of life among university students (Leong Bin Abdullah et al., 2021; Woon et al., 2021a).

### Healthcare System Impacted by COVID-19 and Potential Mental Health Problems

The COVID-19 wreak havocs on the healthcare system. A stable and functional health system should consist of skilled healthcare personnel, well-maintained facilities and infrastructural frameworks, adequate available personal protective equipment (PPE) and medicines, and more importantly, an effective communication with patients (Okereke et al., 2021). The pandemic made a mess of supply chain of necessary healthcare facilities and medicines. Active pharmaceutical ingredients are imported largely from China, India, and the EU, and China is also the biggest exporter of medical devices to the United States. The outbreak of pandemic and implementation of MRPs put the supply chain into risk and contributed to a huge amount of revenue loss (Nicola et al., 2020). Besides, patients reported reduced contact with healthcare providers due to the fear of disease contraction (Okereke et al., 2021). The limited communication between patients and healthcare providers can bring great damage to the otherwise normal healthcare system. Zhang et al. (2020) used a big data approach to measure individual healthcare expenditure and utilization in mainland China. By tracking healthcare utilization before, during and after the Spring Festival in 2020 and 2019, they concluded that total healthcare expenditure and utilization declined by 37.8 and 40.8%, respectively. Due to the pandemic, finite healthcare resources and government grants for healthcare services are allocated to battle COVID-19 (Edoka et al., 2021), thus, many patients have to delay their necessary healthcare such as surgery, dialysis, cancer treatment, physical rehabilitation, and mental healthcare treatment with disruption in services. An investigation established among older adults across 27 European countries found that the prevalence of reported postponed medical care was up to 26% (Ksinan Jiskrova et al., 2021). The sharp decrease in healthcare utilization can lead to turmoil within the healthcare system. Besides, the post-pandemic increase in healthcare facilities admission of indigent patients, medical reimbursement decrease, and high operating costs to main healthcare workers are all contributing factors to the tsunami of healthcare system induced by COVID-19 (Arredondo et al., 2021).

Healthcare workers (HCWs) as primary personnel within the healthcare system suffer from unprecedented burdens due to the pandemic. A narrative review conducted by Giorgi et al. (2020) recognized HCWs as one of the most vulnerable worker categories that may have negative COVID-19-related mental health effects. During the outbreak period of COVID-19, many HCWs applied for going to the frontline to save more lives. However, they had to face a lot of challenges including reducing the spread of infection of COVID-19, developing suitable short-term strategies for patients, and formulating long-term plans to cope with the aftershock of this pandemic. Alshekaili et al. (2020) investigated the healthcare settings in Oman and compared mental health outcomes between frontline HCWs and non-frontline HCWs during COVID-19. They found that HCWs in the frontline group were 1.5 times more likely to report anxiety, stress, and insomnia. The time pressure, increasing workload, and infection risk are all potential “whirlpools” that can drive HCWs to fall into emotional exhaustion, depression, and anxiety (Liang et al., 2020; Que et al., 2020; Wang H. et al., 2021). Even not during the period of pandemic, experiencing high emotional exhaustion has become a daily routine for many HCWs. In the case of United Kingdom, between 31 and 54.3% of doctors reported a high level of emotional exhaustion before COVID-19 pandemic, not to mention the extreme turmoil condition after pandemic (Imo, 2017). Sticking to the frontline work position means the vacancy in the family position. It has been reported that impaired mental wellbeing such as higher severity of depression and psychological stress lowered the social aspect of the quality of life among healthcare workers during COVID-19 pandemic which is believed to be the consequence of mental disturbance and further worsen the already lowered social interaction within the HCW population due to their commitment in battling the pandemic and saving lives (Woon et al., 2021b). HCWs worry about the health condition and infectious possibility of their families but are not able to take action to protect their families directly by themselves. The worries, feelings of guilt, loneliness, and poor sleep quality all contribute to the consistent reports of HCWs’ stress, anxiety, and depressive symptoms (Shreffler et al., 2020; Di Filippo et al., 2021; Repon et al., 2021). Moreover, compared to the other population, HCW were shown to be at nearly three times the risk of infection (Quigley et al., 2021). Even if they are lucky enough to avoid infection, they witness countless infectious cases every day. Hence, it is very easy for them to catch acute stress symptoms or even post-traumatic stress symptoms (PTSS). Yuan et al. (2021) did a meta-analysis on the prevalence of PTSD after infectious disease pandemics in the 21st century and found that HCWs had the highest prevalence of PTSD report (26.9%) compared to the general population and infected patients. A similar review established by d’Ettorre et al. (2021) focused only on the COVID-19 pandemic induced PTSS and reported a rate of up to 73.4% in regarding to the occurrence of PTSS in HCWs. Social support, referring to a high level of social interaction and communication, is believed to be a protective factor for HCW’s mental health wellbeing (Pollock et al., 2020; d’Ettorre et al., 2021; Peng et al., 2021).

### Unemployment Under COVID-19 and Associated Mental Health Problems

In the wake of COVID-19, workers were trapped in precarious employment with a large number of people losing their jobs (Matilla-Santander et al., 2021; Strauss et al., 2021). Pinilla et al. (2021) estimated the unemployment rate in the Spanish employment market in 2020 and found that there was a cumulative increase of 11.9% in the rate of unemployment due to the outbreak of COVID-19. Millions of people, especially those in low-income and middle-income countries, have been pushed into extreme poverty (Barlow et al., 2021). As for the hospitality labor market, business closure policies were associated with a 20–30% reduction of non-salaried workers in the food/drink and leisure/entertainment sectors during March-April of 2020 (Huang et al., 2020). The healthcare sector as the fourth-largest employer in India was reported to face 90% losses due to decreases in out-patient attendance, elective surgeries and international patients, not to mention the healthcare workers individual losses (Gopalan and Misra, 2020). In Hong Kong, the unemployment rate increased from 3.7 to 4.2% and the underemployment rate jumped from 1.5 to 2.1% between January and March (Kaur et al., 2020). An inevitable side effect of losing jobs is the loss of their insurance coverage, which further adds burdens to unemployed families, especially those with patients of chronic disease or preexisting mental health disorders (Arredondo et al., 2021).

Research has established a strong association between mental health and work loss or financial insecurity (Bambra, 2011; Reeves et al., 2016). Sudden layoffs and unemployment due to the pandemic contribute to individuals’ depression, alcoholism, substance abuse, and even in some cases suicides (Brenner and Bhugra, 2020; Rasul et al., 2021; Shi et al., 2021b), not to mention many unemployed status is long-lasting. Not just the unemployed, the underemployed individuals have also been found to have significant psychosocial stress, annoyance, depression, unfriendliness, and insecurities (Kaur et al., 2020). In fact, even though the labor market started to recover rapidly after the peak period of outbreak, the unemployment rate remained high, for example, in the United States, the unemployment rate remained at 6.9% in October 2020 (Yao and Wu, 2021). Bocchino et al. (2021) concluded a new syndrome of the unemployed which is generally devastating for individuals and families. They found a greater symptomatology of the unemployment syndrome in the unemployed compared to the employed as stress, deterioration of the quality of life, low self-esteem, hopelessness, low personal satisfaction, anxiety, hostility, impotence, frustration, sleep pattern disorders, fear, feeling of irritability, and lack of adaptive resources and management of stressors. Besides, the unemployed scored higher in abuse of alcohol, tobacco and other harmful substances, which may further develop into substance abuse without a proper coping strategy (Bocchino et al., 2021). Within all the named symptoms of the unemployed syndrome, losing the meaning of life and individual functioning is the most struggling for many individuals, which is the culprit that can give people the biggest blow (Thill et al., 2020; Levy and Cohen-Louck, 2021; Rosen and Stenbeck, 2021). As employment and personal/household income are the most direct factors related to individuals’ mental health conditions in their daily lives, we summarized existed studies that compared unemployed individuals and employed individuals regarding their mental health problems in Table 2 (Banna et al., 2020; Liu et al., 2020; Patabendige et al., 2020; Ueda et al., 2020; Verma and Mishra, 2020; Hassannia et al., 2021; Munoz-Navarro et al., 2021) and the association between income and mental health problems in Table 3 (Banna et al., 2020; Naser et al., 2020; Que et al., 2020; Shi et al., 2020; Verma and Mishra, 2020; Munoz-Navarro et al., 2021; Zheng et al., 2021a). By comparing symptoms of anxiety and stress between the employed and unemployed, Banna et al. (2020) reported that unemployed individuals were three times more likely than the employed to have anxiety symptoms and two times more likely to have stress (Banna et al., 2020). The likelihood of suffering from depression among the unemployed was shown to be as about twice as that among the employed (Verma and Mishra, 2020).

**TABLE 2:** Summary of findings comparing prevalence of mental health problems between the employed and the unemployed.

References	Site	Participants	Outcomes	Prevalence among the employed	Prevalence of the unemployed	OR (reference: employed)
Banna et al. (2020)	Bangladesh	general	depression	42.3% (153/362)	77.0% (106/138)	/
		population	anxiety	28.2% (102/362)	48.9% (67/138)	4.28 (2.47–7.40)
			stress	56.4% (204/362)	71.9% (99/138)	3.20 (1.97–5.18)
Hassannia et al. (2021)	Iran	health	depression	Physician: 52.0%	41.9% (31/74)	/
(2021)		workers	anxiety	(66/127); Nurse: 51.4% (54/105) Physician: 68.5% (87/127); Nurse: 68.6% (72/105)	59.4% (44/74)	/
Liu et al. (2020)	Turkey	general	depression	26.8% (33/123)	37.0% (10/27)	/
		population	anxiety	10.7% (13/122)	29.6% (8/27)	/
Munoz-Navarro et al. (2021)	Spain	general	depression	9.4% (79/838)	searching for work: 19.6%	1.17
		population	anxiety	12.8% (107/838)	(43/219); not searching for work: 15.0% (28/187) searching for work: 24.2% (53/219); not searching for work: 16.0% (30/187)	1.02
Patabendige et al. (2020)	Sri Lanka	pregnant	depression	18.2% (10/55)	30.2% (55/182)	/
		women	anxiety	20.0% (11/55)	21.4% (39/182)	/
			both depression and anxiety	5.5% (3/55)	9.3% (17/182)	
Ueda et al. (2020)	Japan	general	depression	18.9%	31.2%	/
		population	anxiety	11.7%	25.4%	/
Verma and Mishra (2020)	India	general	depression	19.3% (27/140)	29.0% (62/214)	1.91 (1.07–3.42)
		population	anxiety	22.1% (31/140)	31.8% (68/214)	1.77 (1.00–3.14)
			stress	10.0% (14/140)	12.6% (27/214)	1.25 (0.57–2.72)

**TABLE 3:** Summary of findings exploring the association between income and mental health problems.

References	Site	Participants	Outcomes	Prevalence in low income group	Prevalence in high income group	OR (reference: low income)
Banna et al. (2020)	Bangladesh	general population	depression	55.2% (199/360)	57.1% (254/445)	/
			anxiety	29.5% (106/360)	36.9% (164/445)	1.49 (1.11–2.22)
			stress	53.2% (191/360)	63.8% (284/445)	1.67 (1.18–2.33)
Munoz-Navarro et al. (2021)	Spain	general population	depression	17.2% (43/250)	3.2% (4/125)	/
			anxiety	20.4% (51/250)	12.8% (16/125)	/
Naser et al. (2020)	Jordan	general population	severe depression	/	/	0.50 (0.30–0.82)
			severe anxiety	/	/	0.70 (0.39–1.26)
		health worker	severe depression	/	/	0.38 (0.20–0.71)
			severe anxiety	/	/	0.42 (0.18–0.98)
		university	severe depression	/	/	1.48 (0.81–2.70)
		student				
			severe anxiety	/	/	1.95 (1.03–3.70)
Que et al. (2020)	China	health worker	depression	/	/	0.45 (0.25–0.80)
			anxiety	/	/	1.01 (0.60–1.71)
			insomnia	/	/	0.60 (0.28–1.27)
Shi et al. (2020)	China	general population	depression	/	/	0.74 (0.69–0.78)
			anxiety	/	/	0.75 (0.71–0.79)
			insomnia	/	/	0.91 (0.86–0.96)
			stress	/	/	0.79 (0.77–0.84)
Verma and Mishra (2020)	India	general population	depression	25.9% (15/58)	25.0% (74/296)	0.91 (0.46–1.75)
			anxiety	27.6% (16/58)	28.0% (83/296)	0.83 (0.42–1.61)
			stress	8.6% (5/58)	12.2% (36/296)	0.72 (0.26–2.04)
Zheng et al. (2021a)	China	older adults (age	depression	22.6% (201/888)	16.7% (184/1100)	0.74 (0.57–0.96)
		>50)				
			anxiety	30.7% (273/888)	22.6% (249/1100)	0.68 (0.53–0.85)
			insomnia	28.2% (250/888)	25.7% (283/1100)	0.97 (0.77–1.22)
			stress	23.8% (211/888)	17.1% (188/1100)	0.69 (0.53–0.90)

## Discussion

In this paper, we reviewed the current status of global major economies with the influence of the COVID-19, mental health problems induced by the outbreak, as well as particular sectors seriously damaged by the outbreak and related mental health responses. After comprehensively reviewing existing literatures, we propose a progressive relationship between the pandemic, economic slowdown, and negative mental consequences. In summary, outbreak of COVID-19 induced the imbalance of demand and supply in the world market, and the affected international trade contributed to depressive symptoms of those who rely on international supply chains to earn lives. Similarly, limitation of travel across countries due to the pandemic has put tourism economy into gloomy, and as a consequence, those who work in the tourism industry, who travel to spend their leisure time, or those who travel to earn knowledge and experience cultural exchange would suffer from negative feelings such as loneliness, discrimination, anxiety, depression, and even PTSD symptoms. Impact on the education system of COVID-19 pandemic was mainly reflected through school closure, and associated with quarantine, internet addiction behavior, parents’ working hour contraction, and students’ dropout. The major mental consequences of these changes in economic activities induced by the pandemic were seclusion, panic attacks, comorbid sleep problems, and depression in more serious cases. Healthcare system is another severely vibrated social system by COVID-19. Imbalanced medical resource allocation and declined healthcare utilization is related to negative consequences of relevant economic activities as well as mental health problems of healthcare personnel and patients. Adverse mental experiences of HCWS, especially frontline HCWs, have been extensively studied. Precarious employment was a problem suffered by millions of people following the outbreak of COVID-19. Unemployed and underemployed individuals suffer from not only physical, but also mental hardship.

With the realization of the huge economic turmoil brought by COVID-19 pandemic and so many sectors affected, governments across the globe should take actions to mitigate the economic loss and mental health burden. First, the government should consider its own situation and come up with economic recovery policies targeting at the sectors mostly affected by the pandemic. For example, as face-to-face interaction between healthcare professionals and patients or between teachers and students have been limited, productivity by embracing digital manufacturing technologies can be increased in a more cost-effective marketing strategy in order to keep the balance of healthcare and education systems (Lodha and De Sousa, 2020; Kim and Lee, 2021). Aforementioned MRPs should also be cautiously applied by different governments. Although studies in China showed that isolation-and-quarantine was the most cost-effective intervention in controlling COVID-19, results of studies in other countries can be inconsistent (Rezapour et al., 2021). Therefore, when publicizing strategies to limit virus transmission, the national economic burden as well as individuals’ psychological responses should be considered. Second, we suggest governments or policy makers in different countries to share information and experience in dealing with COVID-19-induced economic slowdown and promote herd immunity worldwide to the *status quo* of the normalization of epidemic prevention. The vaccine can be a strong candidate in controlling the COVID-19 outbreak as well as saving economic loss due to the outbreak as it can help the public to acquire herd immunity in a shorter period which is an effective weapon in fighting against the virus (Sarkodie and Owusu, 2020; Zheng et al., 2021b). Waiting for herd immunity achievement through natural infecting is expected to lose 0.42 years of life per capita compared to the pre-pandemic situation (Gandjour, 2020). A study in the United States suggested that social distancing would not be a cost-effective strategy unless an effective therapy or vaccine could be introduced within 11.1 months of late May 2020 (Thunstrom et al., 2020). Increasing studies have been established and informed the vaccination prioritization (Kohli et al., 2021; Padula et al., 2021; Wang W. C. et al., 2021). Third, stigmatization resulted from misinformation and invalid communication can hinder the application of cost-effective strategies in flattening the COVID-19 curve. Moreover, stigmatization toward mental problems plays a significant and negative role in increasing the psychological burden of individuals with mental health problems, especially in some low-income countries with inadequate mental health services (Kuruthukulangara and Goyal, 2020). In order to minimize the adverse consequence of COVID-19 pandemic and economic shock on the public mental health status, mental health sectors around the world are encouraged to communicate, cooperate and work together when necessary to protect the mental wellbeing of people around the world. We encourage more valid and effective international communications and cooperation in controlling the virus transmission, economic loss, and increased mental health problems induced by the pandemic.

Even though we made efforts to review existing literatures as thoroughly as possible to reveal the economic consequences of COVID-19 and successive mental problems, there are still some limitations in the current article. It is difficult for us to conclude a causal or directional relationship between COVID-19, economic slowdown, and mental health problems. We can only propose a progressive relationship between the three based on current literatures with limited data sources. Besides, we can only display the interrelationship between the three macroscopically. We were unable to look more deeply into the condition of each nation due to the lack of data support in many developing or low-income countries.

Broadly speaking, COVID-19 had induced economic slowdown and adverse mental consequences worldwide with a proposed progressive relationship in between. International trade, worldwide travel, education system, healthcare system, and individual employment condition are major economic aspects that have been affected by COVID-19 pandemic. Loneliness, insecurity, anxiety, depression, sleep problems, discrimination, and substance abuse are adverse mental consequences experienced by individuals experiencing economic turmoil during the pandemic. We call on countries to strengthen cooperation and communication and actively adopt a broad range of economic recovery programs including the adoption of digital and remote trade, education and medical programs and COVID-19 vaccine popularization plan to promote economic recovery to further improve the wellbeing of mental health among multiple vulnerable population worldwide.

## Author Contributions

LL, YB, and SM designed the idea of review and revised the manuscript. YG and XL wrote the draft manuscript. XL, YZ, and HM constructed figures. JQ, KY, WY, and LS reviewed and edited the manuscript. All authors contributed equally to the idea of the manuscript and approved the final manuscript.

## Conflict of Interest

The authors declare that the research was conducted in the absence of any commercial or financial relationships that could be construed as a potential conflict of interest.

## Publisher’s Note

All claims expressed in this article are solely those of the authors and do not necessarily represent those of their affiliated organizations, or those of the publisher, the editors and the reviewers. Any product that may be evaluated in this article, or claim that may be made by its manufacturer, is not guaranteed or endorsed by the publisher.
